# “Eat as If You Could Save the Planet and Win!” Sustainability Integration into Nutrition for Exercise and Sport

**DOI:** 10.3390/nu9040412

**Published:** 2017-04-21

**Authors:** Nanna Meyer, Alba Reguant-Closa

**Affiliations:** 1 Health Sciences Department, University of Colorado, Colorado Springs, CO 80918, USA; 2 International Doctoral School, University of Andorra, Principality of Andorra, Sant Julià de Lòria AD600, Andorra; albareguantclosa@gmail.com

**Keywords:** sustainability, food, environment, sports nutrition, athlete, health, sustainable diet, food literacy

## Abstract

Today’s industrial food production contributes significantly to environmental degradation. Meat production accounts for the largest impact, including greenhouse gas emissions, land and water use. While food production and consumption are important aspects when addressing climate change, this article focuses predominantly on dietary change that promotes both health for planet and people with focus on athletes. Healthy, sustainable eating recommendations begin to appear in various governmental guidelines. However, there remains resistance to the suggested reductions in meat consumption. While food citizens are likely to choose what is good for them and the planet, others may not, unless healthy eating initiatives integrate creative food literacy approaches with experiential learning as a potential vehicle for change. This concept paper is organized in three sections: (1) Environmental impact of food; (2) health and sustainability connections; and (3) application in sports and exercise. For active individuals, this article focuses on the quantity of protein, highlighting meat and dairy, and quality of food, with topics such as organic production and biodiversity. Finally, the timing of when to integrate sustainability principles in sport nutrition is discussed, followed by practical applications for education and inclusion in team, institutional, and event operations.

## 1. Introduction

There is an urgent need to reduce the degradation of natural resources and limit global warming, while providing healthy and sustainably produced food to a growing population. Agriculture contributes greatly to climate change and resource extraction, with animal-based foods playing a major role in greenhouse gas (GhG) emissions, loss of land, water, and biodiversity [1,2,3,4] Further, current dietary patterns contribute to chronic disease through inadequate intakes of plant-based foods and high consumption of red and processed meat [5,6]. In addition, climate change itself will negatively affect food production should temperatures continue to rise, resulting in reduced yields [7,8]—possibly as much as 30%–40% loss by the turn of the century [9]. Adding to this, the consequential sea level rise due to ice melt in the Arctic, displacing not only people but also valuable agricultural land [10], and thus, indicating that food security will likely become the major threat to humans on earth. While agriculture itself must assume more sustainable practices, despite the continued need for intensification [11], strategies for adopting diets with lower environmental impact that are healthy, economically viable, and socially and culturally acceptable are also needed. Thus, for the first time in the history of dietary guidance, food and climate change are crossing paths, and promoting a sustainable, healthful diet, also fit for the athlete, is now more than ever arising as an urgent public and planetary message.

This concept paper is organized in three sections: (1) Environmental impact of food; (2) health and sustainability connections; and (3) application in sports and exercise. For active individuals, this article focuses on the quantity of protein, highlighting meat and dairy, and the quality of food, with topics such as organic production and biodiversity. Finally, the timing of when to integrate sustainability principles in sport nutrition is discussed, followed by practical applications for education and inclusion in team, institutional, and event operations.

### 1.1. Environmental Impact of Food

The environmental impact of food production affects both terrestrial and marine environments. Agriculture uses about one third of arable land, almost three fourths of global water resources, and one fifth of energy. Thus, agriculture is a major contributor to resource depletion [12]. Agriculture also emits large quantities of GhGs. Agriculture accounts for 30% of total GhG emissions from pre-production, production, to post-production [7,13,14], with direct emissions from agriculture contributing the most [7].

Greenhouse gas emissions are quantified in terms of carbon dioxide equivalents (CO_2_eq), collectively also known as global warming potential. Carbon dioxide (CO_2_) is the most prominent anthropogenic GhG with a global warming potential of 1. Nitrous oxide (N_2_O) and methane (CH_4_) are the other two major GhGs, with global warming potential of over 300 and 25 times that of CO_2_, respectively, expressed over a 100-year lifespan [15]. Thus, these GhGs contribute significantly to global warming, and therefore, are at least as important to mitigate as CO_2_.

Direct emissions from agriculture account for the largest fraction in agriculture-related GhG emissions, by generating CO_2_, N_2_O, and CH_4_ directly on the farm [7]. Nitrous oxide arises from fertilizer applied to soil, as part of the denitrification process. Agriculture produces 65% of all N_2_O [16,17]. Methane is generated in large quantities from enteric fermentation and manure from ruminants [1,17,18,19] and, to a smaller extent, from rice production [14]. Further direct, on-farm emissions originate from fossil fuel dependence to run tractors and machinery, which release CO_2_ [7,20]. Adding to this the high demand for animal feed, such as corn and soy, from agriculture, animal agriculture (especially ruminant) plays the biggest role in food-generated GhG emissions and global warming potential [19], exceeding the production of vegetables, grains, and legumes [21,22]. While direct emissions from agriculture contribute the greatest in global warming potential, pre-production processes also include resource-intensive fertilizer, pesticide and herbicide production, which emit GhGs [14]. Climate change mitigation, especially from direct emissions, is critical, as estimates indicate an additional 35%–60% rise in CH_4_ and N_2_O already by 2030 [23]. Table 1 shows GhG emissions per kilogram of various foods.

Food production requires arable land, but there are not unlimited resources. About 33% of Earth’s ice-free surface is used for agriculture [12]. Animal agriculture requires large amounts of land—approximately half of all of agriculture—not only for the animals, but also to produce their feed [1,19,24]. Agriculture has negatively affected the land, with excessive chemical input, causing poor soil health and pollution, with potential adverse human health effects [25,26,27,28,29,30,31,32]. While meat production has become industrial and inexpensive, its impact on animals and people have been largely neglected [19,33,34]. To meet a rising demand for food, especially meat, ecosystems continue to be compromised to clear more land [1,7]. This land clearing is also called deforestation and is an indirect but large contributor to agriculture’s impact on the environment, including the loss of biodiversity [1,2,4].

Post-production GhG emissions include emissions from food storage, packaging, distribution, transport, and end-consumer effects (e.g., waste). Compared to agriculture’s direct emissions, post-production GhG emissions are considered small [14,36]. Taken together, direct (on farm) and indirect (deforestation) effects of agriculture contribute the largest part of all food-related GhG emissions and land use.

Although not always counted in environmental food studies, post-production includes waste. Globally, about one third of food produced is discarded per year [36] with enormous global warming potential [37]. Food loss can occur along the entire supply chain, from harvest to consumer-level discards. The amount of food waste is generally higher in developed countries, although developing nations also show food loss, especially during production and harvest [38]. In developed nations, consumer-level food waste (e.g., households) is significant [39]. In the US, food waste from households has increased by 50% since the 1970s [40]. On average, 40% of food in the US is wasted each year [41]. This amounts to 9 kg of food wasted per person per month or 200 kg of food per 4-person household per year. This has been estimated to cost the American family at least $589 and the entire country $165 billion per year [42]. Food waste is a significant contributor to resource depletion, considering energy, water, and land are needed for production, distribution, and storage of the food that goes uneaten. Moreover, discarding the food adds a further burden to the environment, accounting for 25% of landfill-generated CH_4_ [41]. Thus, besides the energy-costly inputs and GhG emissions from food produced that is unconsumed, wasting it contributes to environmental degradation.

Finally, a significant impact of agriculture on the environment is also its water use. About 70% of all surface and ground water goes to agriculture, with many aquifers showing diminishing reserves [43]. As water resources are becoming equally scarce as land, it is important to consider the significantly greater water footprint of beef production as compared with alternative meat and plant sources [19,21,44], although there are some exceptions [13].

Studies that focus on food and the environment use Life Cycle Assessment (LCA) to quantify global warming potential of the entire food supply chain—from cradle to grave, including all resources used and all emissions to air, soil, and water. While GhG emissions specific to agriculture are commonly reported, comprehensive LCA studies also include land and water use, toxicity to ecosystems and human health, biodiversity loss, eutrophication, and ocean acidification [45]. Although beyond the scope of this paper, the reader is encouraged to consult further literature on this topic [7,13,14,46,47].

### 1.2. Dietary Change to Reduce Environmental Impact

Studies have shown that dietary change can play a significant role in reducing the impact of agriculture on global warming potential, land and water use. Recently, scientists have also linked environmental impact, nutrition, and health in the discourse of dietary change [13,48,49,50]. When considering dietary change as a realistic pathway for the reduction in GhG emissions, land and water use, one of the simplest approaches is to follow healthy dietary guidelines, including a reduction in calories [50,51]. This should not be underestimated since reducing calories, especially if achieved by increasing fruit, vegetables, and dietary fiber at the expense of meat, would result in weight loss and improved health, with enormous impacts on society, including health care cost [51,52,53,54,55].

Animal agriculture is the most costly for the environment [19,21,52]. Eshel et al. 2014 have demonstrated that ruminant (beef) production requires 28 and 11 times more land and water and emits 5 times more GhG, compared with the production of non-ruminant protein sources (e.g., chicken, pork, eggs). Converted to food and protein, beef has a 35:1 feed-to-food caloric ratio compared with a 10:1 ratio for other animal proteins and an 800:1 ratio for feed calories-to-protein ratio, which is almost 10 times lower for other animal protein sources [19]. Thus, eating less beef is becoming an important dietary message worldwide [56]. However, there can be even greater reductions by lowering meat consumption in general [19], and replacing meat, and especially ruminant meat, with plant-based alternatives, which reduces land, energy, and water use, while lowering GhG emissions and waste [21]. While dairy is more efficient than beef, emitting less GhGs, dairy production exceeds egg, poultry, and pork production in land and water use [19,53,57]. Thus, dairy production also contributes to the expansion of cropland and resource extraction which, together with beef production, eventually exceeds the Earth’s safe operating space [58].

Reducing beef consumption and replacing some with plant-based sources, chicken, pork, or eggs could decrease GhG emissions by up to 35% from the food sector []; however, a moderate reduction and replacing beef with dairy has a negligible effect [50]. Replacing beef with fish may provide some benefit but this largely depends on the type of fish, its production system, and fish feed used in aquacultures [3]. Eating less beef can reduce land use by 50% to 70% (see reviews by Hallström et al., 2015 and Aleksandrowicz et al., 2016 [50,59]). Thus, consuming less beef (and dairy) could slow land clearing for feed production and some of this land could be repurposed to grow food for human consumption [53].

Eating less animal and more plant protein in general is also in line with governmental dietary guidelines [56], since most developed nations exceed protein, and especially meat, recommendations [3]. A recent article entitled “Protein production: planet, profit, plus people?” recommends people eat one third less protein overall, replace one further third of their protein intake by plants such as beans, nuts, and grains, and choose the final third from free-range animals [60]. Based on annual per capita intake data [61], if this rule were applied to meat intake, this would still give the average American 80–100 grams (3–4 ounces, oz) per day.

Considering dietary change that could contribute to climate change mitigation, shifting from a typical Western diet to a more environmentally sustainable diet with less meat and more plants would work [59]. Being vegetarian or vegan would be better, with over 30% and up to 70% reduction potential in GhG emissions and land use [21,59,62] and 50% less water use [59]. However, vegetarian or vegan lifestyles may not be preferred for many people [63]. In addition, recent advances also point toward beneficial roles of well-managed, sustainable grazing practices that promote carbon sequestration on rangelands [64], and some areas in the world are less suited for crop production but still provide a great place for livestock, including ruminants. Adding more value to the consumption of meat is necessary [60], however. Thus, in the above example by Aiking (2014), the last third of what used to make up a meat-based dish, should contain a source that can be traced back to its origin, showing a healthy environment where animals are part of an intact ecosystem, given a good life and an end with dignity [60].

## 2. Dietary Guidelines and Sustainability

It is quite clear that eating less meat (especially less red and processed meat), besides eating less overall and more whole and plant-based foods (i.e., vegetables, fruit, nuts, beans, grains), would be one of the most important dietary strategies for both planet and people. These recommendations are also grounded in the dietary guidelines of many countries, some of which have integrated sustainability [56]. 

In recent years, governmental dietary recommendations from various countries have begun to integrate sustainability. According to a recent report by the Food and Agriculture Organization (FAO) [56], of 83 countries that have official dietary guidelines, there are 4 reported countries that reference environmental factors in their dietary guidelines. These include Sweden, Germany, Brazil, and Qatar. Table 2 highlights sustainability commitments beyond those generally targeted to health (e.g., increase plant foods).

The first country world-wide to awaken awareness regarding sustainability and food consumption was Sweden in 2009, calling for a reduction in meat in consumers [65], with a cohort of countries today advising to reduce overall meat consumption to 500 g per week (16–17 oz) [56]. Current meat intake in the US is almost 4 kg (9 pounds, lbs) of trimmed, boneless meat per week, with an annual per capita consumption of almost 90 kg (195 lbs) [66]. However, the US is not alone, as many European and South American countries, along with Australia are also high, but not quite as high. Calculating the yearly per capita consumption per sustainability guidelines, with 500 g per week or a total of 26 kg annually, this equates to about one third of the current US consumption pattern. 

While inclusion of sustainability into the US Dietary Guidelines would have been highly significant, considering (1) the high calorie and meat consumption in the US and (2) the potential global impact of US dietary guidelines [67], it remained invisible in the official guidelines [68]. Although not part of national dietary advice, several countries have published scientific papers that focus on mathematical modeling to derive a regional, sustainable and healthy diet alternative to what is considered the norm. The New Nordic Diet and the Low Lands Diet are two such examples. Both studies focused on less meat, more (Nordic Diet) or less (Low Lands Diet) fish, and local, traditional foods, and both used the Mediterranean diet as benchmark to link health and sustainability [69,70]. Further, there are several quasi-official guidelines, from government agencies or government-funded entities that also include sustainability [56]. Most recently, the Netherlands published an update through the Netherlands Nutrition Centre, calling its citizens to action to reduce red meat intake to less than 300 g per week, while the UK’s governmental agency, encompassing England, Wales, Scotland, and Ireland also added a 7% reduction of dairy products [71].

### 2.1. Are People Willing to Change Diets to Protect the Environment?

When dietary guidelines promote a change, press releases are often the next step, communicating the governmental messages to the public. However, it is well known that dietary guidelines are only marginally followed [72] and that eating behaviors are difficult to change [63], especially if guidelines remain a verbal or written recommendation without the practical skill building required to put the guidelines into practice [73]. In addition, simply telling people what they should eat without communicating the reason behind this recommendation or focusing too much on diet and health may not work either. For example, Hekler and colleagues (2010) showed that a college course on society, ethics, and food changed eating practices more favorably than in students taking courses in health with a focus on biology, obesity, psychology, or community [74]. However, what about sustainability and the environment? Do people (1) understand the link between eating less meat and climate change and (2) would they make the change if it were both good for health and good for the planet? 

“Eat as if there is no tomorrow” [63] studied a sample of Scottish people living in rural and urban areas using focus groups and interviews. The purpose of the study was to examine the perceptions of people toward eating less meat. The authors identified the following common themes, using a qualitative analysis: there was (1) a general lack of awareness related to the link between climate change and meat consumption and (2) little understanding that personal choice regarding meat consumption had anything to do with climate change. Finally, the study also showed that those interviewed were generally resistant to reducing meat intake.

Meat is a traditional menu ingredient in many cultures—meat is often the center piece of the plate. It should be apparent that dietary behavior change will only occur if people begin to understand how best to reduce meat intake. This requires innovative menu design, similar to what has been proposed by the Culinary Institute of America (CIA) and Harvard School of Public Health with the Menus of Change initiative [75], in addition to public campaigns such as Meatless Mondays [76]. This was also the synthesis of a recent study [73], proposing that besides policy change, innovative culinary training through reskilling to cook more balanced vegetarian meals would be necessary. In other words, food literacy training will be needed to bring these ideas closer to consumers.

Regardless of approach taken, promoting meat reduction for personal and planetary health, may continue to be challenging, as was shown by de Boer, de Witt, and Aiking, 2016. These authors studied people’s perceptions as to the extent to which personal dietary change could mitigate climate change [55]. Few recognized eating less meat as an effective way to mitigate climate change, but those who did, showed greater willingness to eat less meat. When asked to rate personal preference of (1) eating less meat; (2) eating more organic food; and (3) eating more local/seasonal food as a vehicle to mitigate climate change, eating more locally/seasonally grown food appealed to more individuals than the other two, including the message of eating less meat [55].

### 2.2. Duality of Sustainability and Health

As we begin to imagine how to integrate sustainability into healthy and athletic lifestyles through the food we chose, we must define what constitutes sustainable food. Sustainability means that “humanity has the ability to make development sustainable—to ensure that it meets the needs of the present without compromising the ability of future generations to meet their own needs” [77] (p. 5). The three pillars of sustainability which include equity, environment, and economics often focus on what is currently unsustainable, for example in food production, but also tend not to challenge the consumer in moving to sustainable development. Sustainability is a moving target, dynamic and ever changing, as the planet is changing. Thus, adapting with the goal to mitigate climate change is needed in all sectors of production, distribution, consumption, and resource recovery, globally as well as locally. While there are numerous examples of sustainable agricultural advances, including organic production [78] and perennial polycultures [79], promoting greater social equity for farmers and welfare for animals and focusing on sustainable consumption patterns are also important. So, what is a sustainable diet? “A sustainable diet is a diet with low environmental impacts which contributes to food and nutrition security and to a healthy life for present and future generations. A sustainable food system is protective and respectful of biodiversity and ecosystems, culturally acceptable, accessible, economically fair and affordable, nutritionally adequate, safe and healthy, while optimizing natural and human resources” [80] (p. 111).

In 2014, Kjӕrgård and colleagues published a framework on how to link sustainability and health and by doing so, dual benefits could be observed [81]. These authors referred to their concept as a duality between sustainability and health. Considering sustainable food choices, a sustainable diet would also be a healthy diet. To understand this duality concept a bit deeper, the example of meat shows the duality of sustainability and health quite well. In general, livestock production, especially beef production, is associated with resource depletion and pollution [4] and contributes significantly to GhG emissions, biodiversity loss, and high health care costs [2]. Excessive red meat consumption has been associated with poor health outcomes, such as cardiovascular disease, obesity, diabetes, and cancer [5,7,82,83]. In addition, how animals are raised in some countries, in confined animal feeding operations (CAFOs), is also of concern regarding animal and worker welfare in the context of community prosperity, health, and quality of life near CAFOs, and human antibiotic resistance from non-therapeutic use of antibiotics in such production systems [84]. The duality of health and sustainability shows that addressing the “eat less meat” message has co-benefits for both sustainability and health. From the consumer side, dietary approaches that promote high protein intakes, such as the westernized diets and those recently termed “the diets of the healthy and wealthy” [85] fail to consider this duality approach, and thus, slow the shift urgently needed to promote both sustainable and healthful eating. There are other examples that provide insight into the intertwined synergies between sustainability and health, such as the ecosystem and health services arising from urban farms and community gardens [86] as well as reducing food waste [87].

Taken together, the inclusion of sustainability in nutrition and health is critical for current and future generations. The science of nutrition must embrace environmental considerations similar to the time when public health nutrition emerged from the science of nutrition for individuals and expanded its reach to communities [54]. “The nutrition of individuals and communities can only be maintained within an environmentally sustainable context, which is currently under serious threat.” [54] (p. 817). Thus, we are in the center of a difficult reality—a sustainable food system needs the support of an intact ecosystem, but the way we currently eat contributes directly to its degradation [54].

## 3. Eat as If You Could Save the Planet and Win!

Whether the intention is health, fitness, and/or sports performance, integrating environmental consciousness when making dietary choices, seems no longer an option but rather a necessity. And while global warming and climate change are often overwhelming topics, dreaming the alternative fires up the imagination of young people, as so beautifully described in the Future of Health by Hanlon and colleagues [88]. Thus, to end a long discourse on the unsustainability of the current food system and its apparent lack to teach us anything about good food and healthy communities or environmental conservation, while the alternative does, the next section will focus on how to integrate these ideas into the daily eating practices of those who train to win. 

### 3.1. Ecological Footprint: This Gets us Thinking

To understand one’s own impact on the environment, it is always a good exercise to calculate the ecological footprint [89]. This is especially true with respect to dietary choices, as most people do not make the connection between their own eating and climate change [63]. However, there are also tradeoffs. Some might travel a lot by plane, which increases one’s footprint significantly. A good tradeoff would be to make sustainable dietary choices to contribute to environmental protection. 

### 3.2. Sustainability in Sports Nutrition?

The integration of environmental nutrition concepts might not be as intuitive in sports nutrition but there are many entry points. One might also argue that due to high energy intakes to meet energy demand, enormous use of packaged foods and bottled beverages, equipment, materials, and heavy travel schedules athletes and their teams should integrate sustainable practices whenever possible.

From a health perspective, athletes lead a more sustainable lifestyle than most of society. Athletes rarely burden the health care system due to chronic disease such as obesity and diabetes. And participating in sports should play a substantial role in making sustainable healthy lifestyle choices. Athletes are also great icons for kids to pick up sports. Athletes are role models for society at large and are generally represented by values of good sportsmanship [90]. Athletes are also great spokespeople, sharing their lessons learned through sports (e.g., time management, discipline to work hard, the importance of rituals, discerning the meaning of failure or injuries). While still dormant, athletes could become a strong voice for planetary health, and begin to realize that success in sport depends, in part, on an intact food system. Athlete or non-athlete, all young people should receive sustainability literacy training, and a covert approach to nutrition education may work best, using experiential learning through taste education, farm visits, cooking and eating together. The conversation around the digital-free table can further the understanding of contemporary food topics and build knowledge surrounding the current issues of the food system and what the sustainable, tasty alternative is all about.

Finally, the sustainable diet is not only the athlete’s responsibility. As we see with other big topics in sport nutrition such as eating disorders [91,92], if coaches, service providers, and administrators are supporting the underlying rationale of a refreshed approach to nutrition education, using sustainability principles, it will enable change. Athletes, coaches, service providers, and administrators all serve as role models for societal change, thus, the adoption of sustainability principles, while coming from the bottom up in our examples, are best diffused if top to bottom is committed and understands the rationale behind the effort. True sport needs true food!

Therefore, let’s get athletes on board in saving the planet and winning! Because sport nutrition is often focused on quantity, quality, and timing of food intake relative to training and competition, below we list sustainability actions for these overall themes, add a section about food literacy and food citizenship for athletes, and consider some final thoughts regarding the integration of sustainable practices for teams, institutions, and international events, such as the Olympic Games.

We will focus on several areas that may apply to exercisers and fitness enthusiasts in general, but athletes in particular, making small steps toward a more sustainable diet as the overarching goal, and this begins with the work of the sport nutrition professional.

### 3.3. Quantity of Food

#### 3.3.1. Eat Less and Better Meat

Athletes’ diets are generally high enough in protein [93,94], if not excessive [95,96]. Current protein recommendations have increased for athletes [97], ranging from 1.2–2 g/kg body weight (BW)/day, especially if the goal is muscle protein accretion [98]. Recently, these recommendations were translated into practical strategies to help athletes maintain protein consumption at intervals throughout the day in the amounts of 0.25–0.3 g/kg BW [99] or about 20 g [100] per meal, given several eating occasions (best every 4 h) [101] per day and before sleeping [102]. This has also been summarized in the recently released position paper on Nutrition and Athletic Performance [97]. That athletes follow guidelines for protein intake is shown in the most recent dietary study on a sample of well-trained Dutch athletes, with mean daily protein intakes of 108 ± 33 g (1.5 ± 04 g/kg BW/day) and 90 ± 24 g (1.4 ± 0.4 g/kg BW/day) in men and women, respectively [94].

There have also been recent trends for even higher protein recommendations to promote health [103], support weight loss strategies [104], to preserve lean body mass (LBM) under hypocaloric situations [105], in resistance-type sports such as bodybuilding [106], and corporate sports performance programs [107]. That athletes, especially in strength and power sports, accomplish higher protein intakes has also been shown [108], with recent reports also highlighting the issue of extremely high protein intakes in some athletes [96,109]. Even though data are limited, practitioners should be well aware of excessive protein intakes in some athletes, aligning with current sport nutrition trends, including the paleo diet. Finally, practitioners may inadvertently promote high protein intakes, considering educational tools and strategies or athletes may simply get too much by eating a lot, since protein is a function of energy intake [110]. However, what are the concerns besides the fact that some athletes may overdo it without proper guidance?

Because this paper is about sustainable diets, the question arises if current protein recommendations for athletes and actual intakes are going to align with global recommendations to reduce rather than increase protein intake in developed countries. Meeting protein recommendations in athletes per se may not necessarily be the issue. The issue is that the continued emphasis on higher protein needs will likely increase the demand for animal protein, including meat, dairy, and eggs. Considering the 50% rise in the world’s population since 2000 and society’s insatiable hunger for meat, the world meat and cheese demand will double by 2050, further burdening the planet [4]. Animal proteins are already consumed in greater quantities than plant proteins, in both the general [66,111] and athletic population [94], and the US considerably exceeds European countries in daily animal protein consumption [112].

Table 3 shows hypothetical amounts of meat (in this case beef) in reference to the (1) non-athlete recommended daily allowance (RDA) for protein [113]; (2) current athlete protein recommendation (~1.5 g/kg BW/day [97]); and (3) recently suggested athlete protein recommendations under energy restriction for weight loss (~2.5 g/kg BW/day [104,105]). It is assumed under this example, that 50% of dietary protein is supplied by meat. This is a rather conservative estimate based on total animal protein intakes typically exceeding 65% in the general population [111].

From Table 3, we can see that meat consumption may easily exceed what is currently considered sustainable, as a total of 500 grams (17.6 oz) of meat per week (~70 grams per day; 2.5 oz) and less or equal to 300 grams (10.6 oz) of red meat per week (~45 grams per day; 1.6 oz/day) would be the upper limit per person. These are also the upper limits for meat consumption of most countries’ dietary guidelines [56], including those for Americans, to promote health [68].

If the recommendation by Aiking (2014) could be implemented it would mean to cut 1/3 of the protein (in this case we would focus on meat, especially red meat), replace 1/3 with plant protein (beans including soy, grain, nuts, seeds), and to choose grass-fed or pasture-raised animal protein sources to obtain higher quality meat with greater omega 3 fatty acids and antioxidants, [114], not to mention less agricultural chemicals and antibiotic residues [60]. 

Let’s look at an example integrating the recommendation by Aiking (2014) from above [60] but with focus on meat, especially red meat. If an 80-kg heavy male athlete eats 120 grams of protein of which 50% comes from meat, it equals approximately 240 grams of cooked meat per day. This is more than 3 times the 70-gram daily benchmark. Thus, if the athlete follows the recommendation for an environmentally friendly protein intake by Aiking (2014), they would first reduce this amount by 80 grams of meat which equals approximately 20 grams of protein [60]. Second, the athlete would creatively adapt protein intake, according to the Protein Flip Initiative (see Table 4), and replace another 80 grams (or 20 grams of protein) by plant sources (See Table 5). The question that will arise is whether the athlete should replace the first third of meat that was cut out, and if so, how would this be done within sustainable boundaries? The answer may be substituting red meat with chicken, pork, or eggs, or choosing a greater proportion of plant-based proteins (e.g., beans, peas, nuts, seeds, and/or grains). However, plant protein may lack essential amino acids (EAA), and thus, may be required in greater amounts to meet the RDA. A recent study compared the land use change and GhG emissions of various animal and plant sources in amounts corresponding to the RDA for EAA [115]. Interestingly, environmental impacts were no longer as discriminatory for animal versus plant proteins, with exception of soy, which showed the lowest GhG emissions and land use. However, we should be cautious when interpreting these data, because people eat a variety of foods in variable amounts to meet daily protein and EAA needs. According to the American Academy of Nutrition and Dietetics Position Paper on Vegetarian Nutrition [116], it is not necessary to get all EAA at one meal, and especially not from one plant or animal. Rather, EAA are accumulated over the course of a day from various foods, and it is not uncommon to find vegetarian meals enhanced with small amounts of animal protein (e.g., dairy, eggs), while vegan meals may include various protein-rich plant foods. Thus, the key message for omnivores is to reduce total amount of animal sources of protein, while for vegans, the message may be to ensure diets meet daily EAA needs by eating sufficient amounts of food, along with a combination of protein-rich, plant-based sources. Working toward a more balanced approach between animal and plant proteins should be the primary goal for both planetary and personal health. Considering the higher protein needs in athletes [97], bugs may be the most suited protein to make up the difference from non-athletic controls, however, at substantially lower environmental cost.

#### 3.3.2. Insects

Insects may well be the next protein source with which excessive meat may need to be replaced. Insects are nutritious, with similar amounts of protein compared to livestock and high levels of vitamins and minerals. Insects can also be a good source of essential fatty acids. Insects emit much lower GhG due to their highly efficient feed-to-protein conversion rates and insects have very low water requirement [117]. Insect powder may become a viable option for post-exercise recovery nutrition in liquid or solid food products, some of which are already on the market. In addition, plant-protein alternatives, such as pea (*pisum sativum*) protein powder, may also present a carbon-friendly source for athletes [118]. Obviously, much more research is needed to compare various plant protein alternatives and insects to the well-researched and popularly used dairy proteins post-exercise. So, what about dairy?

#### 3.3.3. Dairy

Milk, yogurt, Greek yogurt, and cheese all add up quickly, and most Americans, including athletes, may indeed meet the US dietary guidelines, recommending 700 mL of dairy products per day [61]. While milk consumption has gradually decreased over the past decades, cheese, yogurt, and whey intakes have dramatically increased [61]. It is estimated that milk production contributes 2.7% to total GhG emissions [119], although there is great variability based on farming systems [119], with industrial systems generally showing lower GhG emissions due to higher feed digestibility and milk productivity per unit of product, compared to extensive farming systems. However, if other components of environmental degradation (e.g., pollution of waterways and biodiversity), increased energy demand, and human and animal welfare—basically the sustainability of dairy production—are questioned [120], the impact of intensification may well be greater [119].

Globally, about 45%, 20%, and 35% of milk is processed into cheese, milk powders, and fresh or fermented dairy products, respectively [119]. In the US, 50% of raw milk is generally processed into cheese [121]. Milk production generates about 1 kg of CO_2_ eq/kg of milk (or 2.4 CO_2_ eq/kg ready to consume milk) at farm gate [119]. Additional processing, transport, and distribution for dairy products, such as cheese, whey and yogurt increase GhG emissions [119]. Finished products, such as cheese and yogurt, show greater emissions due to the fact they need more milk per unit produced (see Table 1).

Depending on current dairy intake, a climate friendly start could be to reduce dairy products in general, and cheese in particular, due to greater GhG emissions []. The UK [122] currently suggests a 7% decrease in dairy, among reductions in meat, for all citizens to participate in consumer-driven climate change mitigation. This is world-wide the only guideline that targets reductions in dairy. Athletes may want to focus on milk, rich in whey, in the recovery period after an important workout, since this is an effective protocol to promote post-exercise protein synthesis [97] and is palatable. Whether environmental differences exist among milk-derived protein depends on what functional unit is used to express GhG emissions. A Canadian study shows that per gram of protein, GhG emissions are similar or slightly less for cheese and yogurt compared with milk. However, per kg product, milk ranks significantly lower in GhG emissions than cheese and yogurt [123]. Should an athlete need to focus on extra weight/muscle gain, casein-rich Greek yogurts appear popular before going to bed to promote protein synthesis at night [102]; however, Greek yogurt emits more GhGs than regular yogurt, because its production requires more milk [121].

While sweetened yogurts are often loaded with sugar and unrecognizable ingredients, a good choice is the least processed type that contains naturally occurring beneficial bacteria from fermentation. These bacteria are generally known as probiotics and are thought to boost gut health [124]. Thus, for both the environment and health, less processing in yogurts may be the way to go. Because most of the sport nutrition research has been conducted using dairy products, future studies are needed on more environmentally conscious plant protein alternatives and insect protein. This is especially important for athletes who, by default, likely exceed animal protein recommendations from meat and dairy (including whey), currently deemed unsuitable to protect the environment.

#### 3.3.4. Reinventing the Athlete’s Plate

To make the message of meat (and dairy) reduction palatable, practically engaging initiatives are needed. Choosing less and better meat is Slow Food’s global strategy [125] for developed nations, where meat intake is generally very high. Flipping protein on the plate and making meat the topping or side dish is a strategy promoted through the Culinary Institute of America’s Protein Flip initiative [75,126], which originated from the Menus of Change collaborative between the CIA and Harvard School of Public Health. Recreating the plate using meat as a garnish and complementing this dish with whole grain pasta, potatoes, vegetables, and protein-rich grain, legumes, nuts, and seeds is also an easy and creative way to rebuild an athlete’s plate. This is the current topic of ongoing research at the United States Olympic Committee’s (USOC) Food and Nutrition Services, as the Athlete’s Plate [127] was shown to promote more protein than recommended for easy, moderate, and hard training days [128]. Further analysis indicates that the protein dished up on the plates by trained professionals was mostly of animal origin (more than 70%) with marginal amounts of plant protein [129]. It is expected, as was previously shown [73], that food service organization and restaurants may lack the experience with meat-reduced, vegan and vegetarian cuisine. Thus, while flipping proteins of animal-based plates is becoming more popular, taking a closer look at vegan and vegetarian menus and their composition will also help promote plant-based meals for omnivores. Once culinary professionals, students, and nutrition professionals tackle such menus, calculating nutrient profiles could be helpful [130,131], as the outcome of a protein flip menu should not compromise nutrient density—in fact, it should improve it. Most athletes consume sufficient calories to meet micronutrient needs and the majority also takes dietary supplements [132] and eats fortified foods (e.g., cereals, bars), which makes the integration of plant-based eating less concerning. Our preliminary work with the USOC Food and Nutrition Services shows hypothetically that (1) protein flip menus (with less meat) and (2) improved vegetarian menus, increase rather than compromise nutrients, while protein remains at moderate yet recommended levels for athletes [133]. The University of Colorado, Colorado Springs, having transitioned from a corporate to a self-operated dining and hospitality system, recently adopted the CIA’s Menus of Change initiative and serves a very popular protein flip burger at its local food station called “Food Next Door” [134] (see Table 4 for examples).

Protein flip and vegetarian menus provide greater amounts of carbohydrate and fiber [116]. While extra carbohydrates are performance-enhancing, there may be concerns that phytates from fiber may inhibit iron absorption, thus, making the iron from meat less available. One strategy to assist with improving bioavailability of these changed menus is through iron enhancers, including fermented foods. Lactic fermentation is one of the oldest methods for food preservation [135]. Research shows that lactic fermentation of vegetables, corn, and soybeans can drastically reduce phytate content [136], thereby reducing its effect on nutrient absorption. For iron absorption, the mechanism is thought to be through the increase in ferric iron (Fe^3+^), enhancing iron bioavailability [135]. It has also been shown that fermented sauerkraut improves iron absorption [137] and that fermented foods contribute to enhanced nutrient bioavailability in Asian cultures [138]. 

#### 3.3.5. An Omnivore’s Choice to Eat Vegan

While vegan diets may need more caution to ensure protein quantity, quality, and complementarity as well as achieving athletes’ energy availability, it is generally accepted that these diets do not present with adverse health [116] or performance effects [139,140,141,142]. In fact, most data show that plant-based diets are not only great for the environment [13,50], but also human health [13,116,142], and they may promote performance enhancement [123,143]. While some athletes may use vegan diets to mask an eating disorder, there is no evidence that vegan or vegetarian diets cause eating disorders [144]. Considering that a reduction in meat, using more plant-based approaches, is effective in decreasing environmental impact does not mean that athletes must turn vegan. However, integrating meat-less meals and days in omnivorous athletes is not only fun and healthful but it is also educational. Making tasty and nutritionally-balanced vegan meals can also mean a new challenge for those in the kitchen. If proper screening and assessment of individual athlete risk precedes the introduction of plant-based dietary approaches, and education is provided about the rationale for such an approach, there should be no concern.

The best start into an environmentally friendlier diet for athletes is to start right here. As sport nutrition professionals, we need to understand the impact diet has on the environment. Athletes can simply consider the total animal and plant protein contributions in their diet and aim to reduce (not eliminate) red meat first, followed by integration of more plant-based protein choices. A closer look at dairy protein may also be warranted. If everyone in the United States ate no meat just one day per week, it would account for the carbon equivalents of driving 91 billion miles less or taking 7.6 million cars off the road [145]. Reducing meat consumption, in general, can have significant savings overall in food-related GhG emissions and land-use change, exceeding what can be achieved from the transportation sector [50]. Recent research also highlights the individuality of diets and that reductions in environmental impact can be achieved using various approaches, not necessarily compromising personal, cultural, or economic factors [146]. While animal protein reductions in athletes should be of primary importance considering environmental conservation, overall protein intake, nutritional status and the athlete’s cultural background will determine if this is the best approach to take. However, we should not forget to highlight athletes who have been using vegan and vegetarian approaches and athletes who stand up for a healthier environment and restorative farming practices [147].

### 3.4. Quality of Food

#### 3.4.1. Plant Biodiversity—Diet Diversity

In the last 100 years, three quarters of plant and animal species globally have been lost, and the majority of the world’s food supply comes from a dozen plant and a handful of animal species [80]. At the same time, food processing has increased in a way that creates an artificial diversity and a false sense of food security, when browsing through endless aisles in a grocery store. Perhaps it is this level of agricultural simplification that has made the broad field of nutrition oblivious to the topic of biodiversity. Balanced nutrition depends not only on a variety of foods in the diet. The human diet also depends on the diversity within a food crop [148]. While largely understudied and under-documented, fragmented data show vast differences in nutrients within the same species; for example, in potatoes, rice, mangoes, bananas [149,150], and tomatoes [151], but also indigenous corn grown in the American Southwest [152]. Perhaps one of the most striking results in nutrient density comes from the potato, a staple of many countries, and often marginalized as a processed fast food not tolerated on healthy plates. Potato biodiversity is still broad, with over 5000 known varieties remaining and vastly differing nutrient content [150], particularly for sugar, protein, potassium and vitamin C. Similarly, wild plants, still contributing significantly to the health-promoting properties of the Mediterranean regions, have higher amounts of vitamins A, C, and those of the B-complex compared to their cultivated counterparts [153]. Interestingly, wild and local foods are increasingly being recognized as an integral part of contemporary nutrition, as countries are redefining their dietary guidelines, linking sustainable and healthful eating in a traditional context [154,155]. Unfortunately, crop biodiversity and its role in nutrition is generally neglected and this may be due to the field’s professionals [149]. Perhaps a visual comparison as shown in a recent New York Times article [156] brings the message home. We simply assume that a tomato is a tomato and that nutrient density will remain the same despite significant differences [151]. It is true that nutrition education appears to be almost blind to biodiversity [80], although resources are available [157]. Diet biodiversity is becoming a rapidly emerging field but has remained understudied, especially in the nutrition sciences. However, with the return to the farm, scientists are recognizing that agricultural biodiversity can support food and diet diversity, thereby improving nutrition and health [158]. Research is also emerging that agricultural intensification, characteristic of high yield outputs, is associated with the loss of rare plant species [159], but shifting to more sustainable systems, biodiversity may be conserved and ecological functions secured [11]. Losing biodiversity means loss of diet quality, which can lead to micronutrient deficiencies, food insecurity, more pests on farms, fragile ecosystems, and the loss of culture and tradition. Thus, biodiversity should not only be recognized as an important player in sustainable agriculture, but also as a necessary contributor to a healthy diet [160].

#### 3.4.2. Nutrient Composition and Nutrient Density

Dietary choice from the farm or factory gate produces variable foods with variable consequences. Meat and dairy from cows grazing on pastures all their lives provide a nutritionally superior [114], healthier, and safer product [25,52,84], especially considering antibiotic use in CAFOs [161,162]. However, grassfed beef is generally more expensive and considered less sustainable because more land is needed for animals to graze, with greater GhG emissions per kg of beef produced [18]. Unfortunately, animal welfare is not yet part of LCA studies, thus, intensive, as opposed to extensive farming systems, usually fare better in both GhG emissions and land use [18].

Considering the topic of fish, omega-3 fatty acids, especially eicosapentaenoic acid (EPA) and docosahexaenoic acid (DHA) found variably in fish, wild and farmed, have significant health benefits [163] recognized by health organizations world-wide [164]. Fish oils are also popular in athletes [165]. However, fish has been a topic of much debate, not only because of variable omega 3 fatty acid content but also environmental contaminants. Whether farmed fish contains lower, comparable, or greater amounts of EPA and DHA than a wild-caught counterpart continues to be an equivocal topic [166,167,168,169]. The type of feed (plant vs. fish-based) used in farmed fish is one important consideration [170]. Recent trends of vegetable oils in salmon feed have shown to increase the proportion of omega 6 fatty acids, while omega 3 fatty acids decrease [171], potentially impacting negatively on both fish and human health [172]. Although wild fish supply is diminishing fast, if people were to eat the wild fish that are seasonally available rather than the wild fish they desire (e.g., salmon), there would continue to be some level of access—at least for a little while [173]. However, wild fish supplies will not be able to meet the rising demand of a growing world population [3], nor will wild fish necessarily be free of pollutants [168]. Already to date, more than 50% of all fish consumed globally come from aquacultures [3]. Aquacultures generally emit lower GhG compared to wild fisheries, although concerns exist about the feed used in aquacultures [18]. While disconnecting marine resources from fish farming is recognized as an invaluable progress for protecting marine ecosystems, it does not come without increasing challenges about the feed used in aquacultures, especially if produced terrestrially [174]. Perhaps plant alternatives (e.g., microalgae) will be able to provide sustainable solutions in the future, so humans can continue to benefit from fish-derived EPA and DHA.

What about conventional versus organic production? Organic milk provides a superior nutritional profile than conventional milk, with organic milk containing more protein and omega 3 fatty acids [175], and raw milk may provide potential protection against allergies, although there is a greater risk of pathogens [176,177]. On a crop-level, nutrient composition in food has suffered in the last sixty years, with nutrient losses of up to 30%, most likely due to depletion of soil nutrient quality [178]. Conventional agriculture may produce food more economically and in higher quantities. However, research is gradually emerging, showing ecological and human health repercussions of such systems [19,20,21,25,26,29]. Organically grown soybeans show significantly higher nutrient composition, including amino acids, total protein and several micronutrients, compared to conventional and genetically modified (GM) soybeans. While the debate continues whether organic vs. conventional produce is superior in nutrients [78,179], organic foods contain significantly less herbicides, pesticides, toxins, and antibiotic residues [179,180] compared with conventionally produced food. Organic systems can also be more energy-efficient, may thrive in drought conditions, tend not to pollute waterways with synthetic pesticides and nitrate, and typically protect ecosystem services, such as biodiversity (see Reganold and Wachter, 2016, for an excellent review [78]). Finally, while largely understudied considering nutrient content, local food systems provide the most direct pathway from farm to table, with potential for greater nutrient density due to seasonality and reduced transit time from farm to consumer [181].

Taken together, from animals to plants, we must begin to pay attention to the quality of food. In addition, people must understand that dietary choices have the power to either protect or degrade ecosystem and health services. This knowledge may be difficult to teach in a classroom, and it is not nearly as fun as going to the farm. Farm field trips may be especially health-promoting for young, active children, as recent studies show the immune benefit of growing up on the farm [182].

#### 3.4.3. The Grain Chain

The discourse on better food for health of both planet and people, however, is not complete without a discussion on grains. In addition, grains remain the world’s most important staple [80] and may be a key strategy of the Menus of Change initiative. And yet, there has not been more controversy regarding issues of modern wheat [183], including its higher amounts of triggering gluten proteins [184]. Gluten-free eating has seen a tremendous popularity. In athletes, studies show that over 40% of athletes adhere to a gluten-free diet even if they do not have to [185], and despite the fact such diets do not improve performance [186]. However, are all grains as evil as they sound? 

First, whole grains are packed with fiber, protein, carbohydrate, and B-vitamins and studies show whole grains reduce all-cause mortality and morbidity, with lower risk for cardiovascular disease, cancer, and diabetes [187]. When wheat is grown organically it has been shown to contain superior nutritional profiles compared to conventional wheat [188].

Wheat’s nutritional profile has significantly decreased since the 1960s [189,190,191], while the number of new gluten proteins has increased [192], and this is reportedly not due to changes in soil, but changes in wheat hybridization. On the other hand, ancient wheat such as einkorn, emmer, kamut, durum, and spelt, celebrating a recent comeback, exceed nutrient composition (e.g., protein, lipids, minerals and elements, antioxidants such as lutein) compared to modern wheat [190,193,194,195]. A recent study on khorosan, also known by the name Kamut, shows greater anti-inflammatory effects through antioxidants, blood minerals, and reduced metabolic (e.g., lipids, glucose) and oxidative stress markers in healthy subjects consuming khorosan in bread, pasta, and crackers for 8 weeks, compared to consuming these products made with a semi whole-wheat product [196]. Thus, these older cultivars may contribute significantly to nutrient dense diets [195] and health promotion [196].

Such ancient wheat varieties are not only more nutritious and promote antioxidant protection, but some also lack the highly immuno-suppressive α-gliadin peptides—the major component of gluten that provokes gluten intolerance. These are encoded by the D-genome of wheat. Thus, species that lack the D-genome of wheat, such as einkorn, emmer, and durum, show lower reactivity compared to common wheat [197]. Work in Italy is currently focused on einkorn, the oldest form of wild wheat first domesticated 12,000 years ago by hunter-gatherers in Mesopotamia. Along with wild emmer, also known as the mother of all wheats, “einkorn is considered a catalyst of agriculture and the initiation of wheat’s vast biodiversity” [198] (p. 22). Einkorn appears to either pose no [199] or fewer adverse reactions in Celiac patients compared to modern wheat [200]. Athletes who have been diagnosed with Celiac’s disease, should consult their sports dietitian before trying einkorn since it may still have the potential to induce the Celiac’s disease syndrome [201]. For a great review see Kucek, Veenstra, Amnuaycheewa, and Sorrells, (2015) [202].

Understanding nutritional differences among grain varieties also opens the dialogue on bread. While the choice of grain permits greater nutrient intake, fermentation using a sourdough starter has also been shown to increase bioavailability of nutrients such as iron. This is most likely due to a reduction in phytates [135]. Fermented bread decreases post-prandial glycemic response through organic acids that delay gastric emptying [203]. This, therefore, is a great low glycemic alternative to processed white bread for active individuals, especially at breakfast. Finally, sourdough fermented bread also appears to retain antioxidants better due to lower pH levels [204], which if baked with an antioxidant and protein-rich grain, such as einkorn or emmer [193], by far exceeds the nutrient density compared to bread made with modern wheat. 

Studies also show that both germination (sprouting) and fermentation (e.g., sourdough baking) can break down gliadin, one of the gluten proteins known to increase reactivity. While still not safe for Celiac patients [205], there are fewer immunoreactive peptides in sprouted products [202]. As for fermentation, lactic acid bacteria degrade some of the gliadins but multiple microbes appear to be needed to effectively degrade the majority of gliadin [206]. In a study by Greco et al., (2011), 97% of gluten was degraded by fermentation [207]. However, there were still a few Celiac patients in the study who showed measurable villi atrophy compared to non-gluten control treatments. Thus, wheat sourdough fermentation, as compared to non-fermented flour, does not degrade gluten enough to prevent adverse responses in Celiac patients [208].

Taken together, there does not seem a clear relief for Celiac patients, from ancient or heritage wheat, whether sprouted, fermented, or not. However, research suggests that there may be wide variability among reactivity to gluten, depending on the type of grain and level of processing. In addition, Celiac’s disease expression, while triggered by gliadin-induced antibodies, can also be quite variable. While Celiac’s disease has become more prevalent, with about 1% of the general population being affected, only 10%–20% of people appear to be aware of their condition and follow a strictly gluten-free diet [209]. Mild forms of Celiac’s disease, however, have the potential to worsen with age, thus, management through a gluten-free diet is necessary to decrease severe complications, such as osteoporotic fractures and intestinal cancers [209].

Interestingly, there are also other clinical presentations that do not fully correspond with Celiac’s disease but rather consist of new clinical syndromes, typically termed non-celiac gluten and/or non-celiac wheat sensitivity. Though controversial and under-studied, it is generally accepted that these syndromes exist, but in the absence of gluten-ingested, celiac-specific antibodies [210] or wheat allergies [192]. What ultimately triggers these syndromes is unclear, as it may not need to be gluten but could include other components of wheat, such as the low fermentable, poorly absorbed, short-chain carbohydrates (FODMAPs) [211], other proteins [212], or insecticides such as glyphosate [29,30].

Regardless of exact mechanism, variability in clinical symptoms from grain or wheat ingestion pose new opportunities for nutrition professionals, considering both, the recent changes in clinical presentations and the modernization of many plants, including wheat [192]. While challenging, this should provide new avenues for dietary management of those who prefer a gluten-free diet for performance enhancement or health promotion, in the absence of Celiac’s disease, to trial various approaches [192,207,213,214], as opposed to eating a strictly gluten-free diet. A gluten-free diet per se, with a high amount of processed gluten-free foods, may not meet nutritional recommendations, and a gluten-free, vegan diet could pose serious negative health and performance effects (e.g., B-vitamin deficiency). It has also been suggested that individuals should choose grains and their processing wisely, as this may reduce the risk of developing Celiac’s disease in those who may have hereditary risk [202]. As ancient and heritage grain production is sweeping through the United States as a long-awaited player in the local food movement, the grain chain, from farmer to baker to table, is filled with food literacy opportunities for athletes such as making bread together. After all, bread has been a staple around the world with thousands of traditional uses. Bread is also one of the primary carbohydrate choices for athletes in training and competition, and carbohydrate is the major source of calories for most humans [113], including athletes [215].

While ancient and heritage grains are making their way back to the grocery stores, their production remains relatively small. However, these grains are known to be more drought tolerant [198] and using grains in crop rotation or as cover crop can meaningfully contribute to farm diversification and sustainable agriculture [78]. Grain production may soon take a turn for the better and become more sustainable as scientists at the Land Institute in Salina, Kansas [216] will likely announce that perennial grains (long roots capture carbon, enhance soil quality, and help reduce erosion) may replace modern wheat, not only on the field but also in people’s bread baskets. 

### 3.5. Food Literacy and Food Citizenship in Sports and Exercise

There is much to relearn when it comes to food. Perhaps we have moved away too far from field, farm, and the kitchen to know where food comes from and when it is in season. We also have lost important life skills such as cooking. We have to relearn and teach these simple skills to rebuild the knowledge needed to establish a healthy relationship with food. This brings us to the topic of food literacy. Recently, Vidgen and Gallegos, 2014 defined food literacy as the following:

“Food literacy is the scaffolding that empowers individuals, households, communities or nations to protect diet quality through change and strengthen dietary resilience over time. It is composed of a collection of inter-related knowledge, skills and behaviors required to plan, manage, select, prepare and eat food to meet needs and determine intake. This can simply be translated as the tools needed for a healthy lifelong relationship with food” [217] (p. 54).

Academic programs that promote food literacy, through curricula that meet joint goals of health promotion and sustainable development, especially in the health professions, may allow for transformative experiences [74]. Such food literacy discourse has the ability to diffuse, with outcomes that promote food citizenship in young people, and therefore, future generations [218,219]. Food citizenship is the practice of engaging in food-related behaviors (defined narrowly and broadly) that support, rather than threaten, the democratic, socially and economically just, and environmentally sustainable food systems [220] (p. 271).

Athletes and their support staff should be introduced to the link between daily food choices, health, and sustainability. It is most likely the sports dietitian who will bring this topic to the table, and the best and least confrontational approach, may be through a sustainably sourced meal cooked together such as a “Team Dinner” or a fun food literacy event with multiple stations, competitive team work, and food-related prizes. Shopping at local food outlets, including the farmer’s market, and cooking together might be other options to open the dialogue pertaining to sustainable quantity and quality of food, as discussed above. Eating practices and fueling strategies are a performance-determining factor; however, becoming a food citizen [221] with knowledge and skill to navigate through an ever more complex food web opens the narrow sports-performance focus of a young athlete and introduces areas such as environmental conservation. Thus, sport nutrition education should begin to integrate sustainable food topics and promote food citizenship and food literacy by an enabling, participatory approach when the timing is right and where opportunities arise.

#### 3.5.1. Athletes to Farm

While sport nutrition is a broad field and athlete performance and health issues take precedent over sustainability efforts, the sports dietitian will need to find a good balance that allows for sustainability integration, without feeling constrained but rather enabled in promoting awareness, building knowledge, and enhancing skills around food, ultimately improving dietary habits of young people. Thus, going to a local farm and/or market to buy food is only the first stop in this refreshed sport nutrition curriculum. While athletes often crave for the latest in exotic products from far away (e.g., Acai berries), eating some of the unfamiliar and wild foods grown close to home, may not only be more nutritious, but will also come with a plethora of learning opportunities. To allow athletes to make a connection with their home environment through the farmers who grow their food, training plans may need to be flexible to allow for Community Supported Agriculture (CSA) share pick up, a farmer’s market visit, or a farm-field training day, as this may offer invaluable experiential nutrition education. The opportunity for athletes to experience “local life” is short but is increasingly meaningful, as sport teams and elite athletes are in the spotlight at home. Engaging with the local community may bring personal and team-related benefits for farm-fresh food support that has the potential to strengthen athletes’ community involvement and build a sense of place. Finally, investing in the community, through food procurement from local farms, may also set the precedent for a supportive environment should athletes get injured or to facilitate the transition from athletic to normalized life after the career is concluded.

Eating locally grown and raised food has many benefits, but it may not automatically be more environmentally sustainable. Nevertheless, the local food movement might ignite people’s desire for better taste, connection to place and to the people in their community. Local food seems to attract people also because of its economic benefit to the community, and there is a general sense, despite the fact that local food often costs more, that it is more affordable [222]. Regardless, those having worked and experienced the local food movement cannot let it go, and while the urgency to become more food secure in this changing world calls for revolutionary action through more sustainable food production [11], engaging in local food mobilizes people on a deeply emotional level, often difficult to express for those who are in it [223], but likely the reason why people may identify it as a realistic way to change eating behavior [55]. Recent research also shows that those buying direct from the farmer think and act around food very differently, compared to those going to a chain grocery store to procure their food [221]. Thus, the local food system is engaging inter-personally and economically within a community and it also teaches about food, the seasons, biodiversity, flavors, nutrition, cooking, culture and tradition.

While not always the most sustainable, the local food system may be a vehicle that could direct people to healthier and more mindful eating. A recent study illustrates how the awareness of dietary choices and eating can meet joint goals of individual health, environmental sustainability, and food security [224]. Thus, the many facets of a local food system can act as living learning laboratory to practice mindfulness training, even in athletes and their teams, as they cultivate both eating for sport and eating for planet Earth.

Local food systems are defined as “collaborative effort in a particular place to build more locally based, self-reliant food systems and economics—one in which sustainable food production, processing, distribution and consumption is integrated to enhance the economic, environmental and social health of a particular place.” [225] (p. 100).

If athletes receive food money, a resource factsheet with local food procurement options could begin the collaboration with local business. A factsheet could also promote best choices when shopping at grocery stores, how to identify what’s locally produced, what’s seasonal, which labels to observe (e.g., USDA Organic; Buy Local; Marine Stewardship Council, MSC or Aquaculture Stewardship Council, ASC; GMO Free Project; Humanely Raised, American Grassfed, Direct or Fair Trade), the list of the dirty dozen [226], and how to order in bulk online, including heritage/ancient grains. Identifying farm-team partnerships requires farm visits and direct communication with the farmers [227]. In addition, providing some community service at the farm with 1 or 2 workouts held at the farm per year, supporting planting, weeding, or harvesting, will facilitate access to local, farm-fresh food because a connection is built much to the delight of the small-scale farmer who feels supported by the local sports team. Teams may also obtain group discounts if ordering in bulk through local buying clubs, food hubs, or food cooperatives. With CSA shares, there is great flexibility should shares get temporarily suspended when athletes travel. It is also possible to obtain surplus food and getting parents involved to preserve this food for later. Preservation, including fermentation, will not only support nutrition programming, but could be applied at times of increased team stress when athletes’ immune function is more susceptible to illness. Locally, seasonally, and organically grown produce is more nutritious [175,179,180,188,228] and fermentation (e.g., pre- and probiotics) may add immune [229,230] support in times when athletes need it. Check with local University Extension offices for safe guidelines on canning and fermentation.

#### 3.5.2. Taste Education and Cooking

Taste education with athletes can be integrated at any time, combined with a general team talk (locally grown fruit, vegetables, or grains as tasters), fueling or recovery workshops (integrating seasonal fruit, yogurt, and honey), or even during a travel nutrition talk (cultural food tasting of the travel destination). Nutrition should no longer be taught without hands-on learning from farm to kitchen. Written or visual materials (e.g., posters) or recipes that integrate local producers, topics of food citizenship (e.g., farmer’s market shopping), or health benefits of diet diversity (e.g., biodiversity of greens) will keep building awareness and return home economics to young people’s lives. Edible nutrition education is not only fun, inspiring, and tasty but it also teaches young people important skills and it builds a lifelong healthy relationship with food—and that is food literacy [217]. In addition, working with a farm-to-training table curriculum in sports also provides an opportunity to highlight local producers, dairies, farmers, or bakers, the history of the place, and this brings meaning and relational values [231]. If time is tight, University nutrition programs may partner to support a revisited curriculum that integrates agriculture and culinary training. One such example is the Flying Carrot Food Literacy Truck [232]. This program has been led by graduate students in sport nutrition at UCCS for the past 5 years [233]. After initial inception, several food-related courses, internships, and service learning experiences within the Southern Colorado regional food system, including a campus farm with its farm-to-table café, Food Next Door [134], and local food literacy farmhouse, are now serving a vigorous on-farm and in-kitchen curriculum for undergraduate and graduate students at UCCS, some of whom are in sport nutrition.

#### 3.5.3. Budgets, Planning, and Food Waste

Food literacy should also integrate the full circle of engaged eaters’ choices, including the discussion of food waste. Athletes and their families may tap into rescued food programs if budgets are tight. Most cities today have food rescue programs and some cities and programs, such as the one known as P.O.W.W.O.W. by the Borderland Foodbank in Southern Arizona [234], have made it possible to access fresh food, at affordable price, that otherwise would go to landfill. Food waste at the consumer level originates especially due to consumers’ aesthetic preferences and arbitrary sell-by dates [36] as well as simply by purchasing, cooking, preparing and serving too much [39]. Teaching athletes to purchase what they can eat, cook what they purchase and promoting safe preservation and freezing techniques, are all part of food literacy training. The sports dietitian can help with weekly planning, providing input with shopping and cooking, so that athletes learn when, what, and how much to cook and to plan their dietary strategies, as much as the coach plans their training schedule. Cooking and planning ahead has been identified as a critical strategy to reducing food waste on the consumer level [39]. When traveling, a little bit of research ahead of time will pay off. Food cooperatives often have restaurants and there are many “Pay-What-You-Can” non-profit community restaurants in the US that serve local and organic food, often rescued from what would have otherwise been wasted, and sold at very low price (or what the team can pay [235]). These types of food outlets, including food “waste” supermarkets, are becoming more available everywhere. Obviously, each such stop will add to nutrition education and athletes learn they can eat this way everywhere they go. For good restaurant, market, and café guides that serve local, sustainable, and organic food, see Slow Food USA or Edible Communities [236,237].

### 3.6. Timing of Sustainability Integration in Exercise and Sports

In this paper, we addressed the environmental impact of food choices, easy changes that can be made (e.g., eating less meat), and paying attention to the food value chain to obtain high quality food with zero waste strategies. We have also integrated the local food system as a great entry way to connect sustainability and health, leveraging co-benefits for both planet and people. Posing the question on when to integrate sustainability principles in sports nutrition may sound as if athletes and sport teams have a special status concerning the food of the future. The answer is, nobody does, and shifting to a low-carbon consumer culture is a necessity rather than a choice. However, there needs to be careful consideration when to launch or what to initiate within the economic boundaries of grass-root sports, where parents are the coaches and kids are running from A to Z with plastic wrappers in their hands, squeezing out their pre-game meal. Likewise, timing considerations on the elite level must involve everyone because the budgets will have to, at least in part, account for increased food costs, cooking and team dinners, and time to pick up fresh food at farmers’ markets, farm stands or neighborhood stores. 

The best timing to plan any new programs within the world of sports is usually as the season is coming to an end and early before the start of the next training cycle. This is especially true should extra resources be needed to support the program. Farm CSA shares cost between $500–$600 for 6–8 months or about $20–$30 weekly, with each share providing food for about 4 people. Team talks with edible tasters will either require planning and connecting with local producers for samples or more expensive transactions at the store or market. Thus, the more time is invested to form farm-to-sports partnerships, the better and more economical the outcome.

All athletes spend time training at home. This is the best time to teach shopping and cooking. Depending on the season, it is also the best time to introduce local food with farm and market visits. Even though athletes are still on the go every day while in training, there is the potential for community connections through the local food system. Thus, providing a platform for this to occur may create a new sense of purpose, external to the identity of being an athlete. Participating in the community may balance the lives of the elite and new friendships may arise with those who work the land, which may create awareness of earth stewardship and food citizenship.

Once a program launches it is difficult to hold it back and it will evolve on its own. This is especially true for the local food movement. It needs ignition, but once the web is being explored and experiences are made, there is no going back. It is a paradigm shift. It’s a local food revolution [223].

Should sustainability be a topic while traveling? The answer is yes, because in many countries, sustainable food systems are still the norm. Thus, traveling to European and Eastern European countries is often an eye-opener. Taking athletes into the grocery stores or through a local market is food literacy away from home, and sports dietitians also increase their knowledge and skills when exploring foods abroad. While most travels abroad are hectic with little time, surprisingly, the Olympics may be the perfect place for food literacy. The local volunteers are a great resource for information and they provide access to local markets to purchase fresh food. Thus, even when traveling, there are multiple opportunities to broaden food experiences and teach important cultural food differences.

Finally, introducing sustainability in sport nutrition may also be timely for those who are injured. These athletes may have more time and interest to learn about whole, nutritious food and cooking that could enhance healing. In addition, introducing athletes to other areas outside of sport, such as agriculture or cooking, may distract the overly occupied mind, and help maintain a positive attitude during the recovery and return-to-play period.

Taken together, while the timing of sustainability integration must be carefully considered to bring change to nutrition programming for athletes, small steps can fit everywhere and they bring with them deliciousness, beauty, and inspiration to participate in the food chain from farm to kitchen and table. There should be no doubt that this is the future of how nutrition should be taught, also in sports.

### 3.7. Integration of Sustainability Practices as Collective Commitment in Sports

#### 3.7.1. Team Sustainability

Integrating sustainability in the sport nutrition program benefits first the athlete. However, coaches and other members of the sport science team, including athletic trainer, sports medicine doctor, and psychologist all benefit. Because of the performance enhancing team approach and multi-disciplinary strategies, sustainability and food will also open the dialogue of sustainable practices in general. This may mean that the team develops a vision or even a policy for sustainable development, especially considering training venues at home, where more influence is possible. Starting with food and drink, this may mean the team implements a recycling, re-using, and composting strategy. It may mean the team bans bottled water and throw-away, take-out containers. And it may mean preferred vendors for training tables or team meetings come from local businesses, using sustainably and locally sourced food. Catering may be enhanced through the-less-but-better meat initiative in combination with highly nutritious grains and beans, seasonal vegetables and fresh fruit. Team commitments may also include coach and support staff’s eating practices that are coherent with the underlying philosophy of eating for performance and health. Finally, taking on a team vision for a sustainable future may also inspire parents and families and this could be supported by social media and website resources. A great example of how sustainability can be part of every sporting venue is the Green Sports Alliance [238].

#### 3.7.2. Institutional Sustainability

Whether it is at a high school, university, or national/regional/local sport center level, integrating sustainable food procurement into food service starts to open many opportunities. It allows for a new seasonal menu. Reducing meat through the protein flip and boosting vegetarian offerings, sparks creativity in chefs and curiosity in athletes. Sourcing locally brings in the story of the farmer, unknown diet diversity, and awareness related to the link between fresh food and health on an individual, community, and environmental level. If institutions have gardens or farms, there is potential to integrate edible education linked to the menu served, in addition to the invaluable seed- -to-plate menu. However, change is always more challenging than we think. Thus, to initiate a new menu, it is crucial that athletes and coaches understand the rationale behind the change. If resistance develops, athletes could be integrated in various educational activities that incorporate their own food preferences, cooking competitions, or recipe contests. It is helpful for athletes to see protein numbers of a meal and over a day to reduce fear of not getting enough. From a health perspective, there are many opportunities when food service commits to a more sustainable menu, with procurement gradually shifting to seasonal, organic, local, pasture-fed, free-range, and sustainably produced, fished or farmed food.

#### 3.7.3. Event Sustainability

Integrating sustainable food into sporting events is being done on many levels. Some examples include London 2012 [239] and Rio 2016 [240]. Both local organizing committees published their sustainable food visions and made procurement with sustainable agricultural standards a priority. Especially the Rio Games were impressive as to the portrayed commitment to environmental consciousness through sustainable sourcing, improving supply chains, managing packaging, and reducing waste. As previously discussed, Brazil is one of the few countries whose governmental guidelines have embraced sustainability [56]. Whether visions and guidelines are ultimately implemented at the international events is difficult to tell, as there has been no labeling that details sustainable sourcing. This has previously been noted and published by Pelly et al., 2014 based on a survey conducted by sports dietitians, representing various countries at the 2012 London Olympic Games [241]. While the international sport nutrition organization, Professionals in Nutrition for Exercise and Sport (PINES) [242] reviews the menus for each Olympic cycle, an on-site implementation phase could help improve both menu and labeling, with inclusion of sustainable sourcing. In addition, the athlete dining hall and the Olympic village present an enormous challenge to sustain environmental commitments, considering food waste, bottled beverages, and to-go meals. In the future, food service at the Olympic Games should promote sustainability more visibly, highlighting a country’s food culture and offering athletes experiential learning opportunities that showcase regional food traditions, seasonality, world heritage, and the story of farmers. Tokyo 2020 would be an excellent host city to bring change to the athlete dining hall with greater transparency for sourcing, local food literacy, and hands-on learning (e.g., how to make tofu or soba). The Olympics are long and many athletes have downtime. Why not learn something about the host country’s food culture, sustainability efforts, seasonality of food, and how traditional foods are produced? While currently implemented at the Youth Olympic Games, integrating the host country’s food traditions could augment the cultural experience of athletes visiting the Olympic village dining hall.

## 4. Conclusions

Environmental impact of food production is high, especially when considering the GhG emissions, land, and water use of animal agriculture. Many governmental organizations are beginning to integrate sustainability into their dietary guidelines and are calling on consumers to eat less animal and more plant-based foods. Integrating health and sustainability creates co-benefits, as for the most part, sustainable eating also means healthful eating. Nutrition recommendations, for active and athletic individuals should also begin to integrate sustainability. Using innovative approaches, including experiential learning from farm to table, renews the relationship of food by rediscovering the broad meaning of food, building knowledge and skills in the kitchen, and sharing food around the table. Initiating sustainable practices in sport, including sustainable food procurement, opens many opportunities for athletes and their entourage to engage in local and regional food systems, and by curbing the appetite for meat, individuals, teams, institutions and organizers begin to contribute to a reduction in global warming from the food sector.

## Figures and Tables

**Table 1:** Greenhouse gas (GhGs) emissions in food.

Low GhGs	Medium GhGs	High GhGs
1 kg CO_2_ eq/kg edible weight	1–4 kg CO_2_ eq/kg edible weight	4 kg CO_2_ eq/kg edible weight
Potatoes	Chicken	* Beef
Pasta	Milk, butter, yogurt	* Lamb
Bread	Eggs	Pork
Oats and other grain	Rice	Turkey
Vegetables (e.g., onions, peas, carrots, corn, brassica)	Breakfast cereals	Fish
Fruits (e.g., apples, pears, citrus, plums, grapes)	Spreads	Cheese
Beans/lentils	Nuts/Seeds	
Confectionary	Biscuits, cakes, dessert	
Savory Snacks	Fruit (e.g., berries, banana, melons, salad)	
	Vegetables (e.g., salad, mushrooms, green beans, cauliflower, broccoli, squash)	

* May be as high as 20–50 kg CO_2_ eq/kg edible weight. Average CO_2_ emissions for driving car are 0.186 kg CO_2_ eq/km driven. Adapted from [35] (with permission).

**Table 2:** Sustainability commitments in Germany, Brazil, Sweden, and Qatar.

	Germany	Brazil	Sweden	Qatar
Sustainability Highlights	Eat meat in moderation. Use fresh ingredients. Take your time and enjoy eating. Eat fish once or twice a week.	Choose seasonally and locally grown produce. Try to restrict the amount of red meat. Limit the amount of processed foods. Eat in company. Develop, exercise and share cooking skills. Plan your time and make food and eating important in your life.	Eat less red and processed meat (no more than 500 g of cooked meat per week). Choose eco-labelled seafood. Try to maintain energy balance by eating just the right amount.	Limit red meat to 500 g per week. Avoid processed meat. Eat less fast foods and processed foods. Build and model healthy patterns for your family. Eat at least one meal together daily with family.

**Table 3:** Daily protein recommendations using estimated daily meat contributions for athletes and non-athletes.

Example	Units	Non-Athlete PRO RDA	Athlete‘s Standard PRO	Athlete‘s Hypocaloric PRO
60 kg female	PRO (g/day)	48	90	150
	Cooked Meat Contribution as 50% of total PRO (g/day) *	92	172	288
80 kg male	PRO (g/day)	64	120	200
	Cooked Meat Contribution as 50% of total PRO (g/day) *	123	230	387

* meat contribution at 50% of total protein recommendation, calculated for cooked ground lean beef (15% fat); 100 g edible portion equals 26 g of protein (similar for chicken, pork, lamb). Athlete's standard diet calculated at protein recommendation of 1.5 g/kg/day [97]. Athlete’s hypocaloric diet calculated at protein recommendation of 2.5 g/kg/day [104,105]. PRO = Protein. Most sustainable and healthy dietary recommendations target 300 g of red meat or 500 g of total meat per week [56]. RDA = Recommended Daily Allowance. Table shows how easily athletes may exceed these weekly meat recommendations if they ate 50% meat of the total protein recommended per day.

**Table 4:** Examples for protein flip menus and burgers.

Meal	Actual	PRO g	Protein Flip	PRO g	Comments
Grilled Beef with Quinoa and Veggies	4 oz beef	26	2 oz 100% grassfed beef	13	Rename to Southwest Anasazi Bean and Beef Bowl.
United States Olympic Committee	4 oz kale and quinoa	4	4 oz kale and quinoa	4	Launch educational campaign on protein flip.
Colorado Springs	4 oz broccoli	3	2 oz Anasazi beans	10	Add history of Colorado beans and quinoa.
	1/2 stuffed portobello	5	4 oz broccoli	4	
			1/2 stuffed portobello	5	
	total	38	total	36	
Pork loin with Poblano Chili and Rice	4 oz pork loin	26	2 oz organic pork loin	13	Rename to Ancient Grains with Poblano Chili Pork.
United States Olympic Committee	4 oz poblano chili	3	4 oz poblano chili	3	Launch educational campaign on protein flip.
Colorado Springs	4 oz white rice with veg	4	6 oz farro, beans, veggies	12	Integrate nutritional benefits of ancient grains.
					Add history of emmer and biodiversity of grains.
	total	33	total	30	
SWELL Burger	4 oz beef burger	22	2 oz 100% grassfed beef	10	This meal is served at UCCS Food Next Door.
University of Colorado	white bun	5	1.75 tsp black beans	2	SWELL Burger uses the protein flip approach.
Colorado Springs	1 cup dinner salad	1	1.75 tsp quinoa	1	Launch educational campaign on protein flip.
			1.75 tsp hemp	3	Integrate sustainable food literacy.
			1 T peppers, carrots, leeks, chard	1	Highlight nutritional benefits of grassfed beef.
			garlic, chili, cumin, chives		Include social justice issues regarding CAFO.
			1 slice socca (chick pea flatbread)	4	Highlight Slow Meat and Menus of Change ideas.
			SWELL kale salad with roasted veg	2	
			pumpkin seeds	2	
	total	28	total	25	

SWELL: Sustainability, Wellness, Learning; UCCS: University of Colorado, Colorado Springs; PRO: Protein; CAFO: Confined Animal Feeding Operation; ounces (oz; 1 oz = 28.4 g); tsp: teaspoon; T: tablespoon.

**Table 5:** Cooked amounts of plant and animal-based foods delivering 20 g of protein.

Food	Grams	Ounces	Cups	T	Calories	Limiting Amino Acids	Leucine (g)
Anasazi Beans	322	11.4	1.4	23	426	Sulfur containing AA	1.2
Black Beans	295	10.4	1.3	21	295	Sulfur containing AA	1.3
Chickpeas	284	10	1.3	20	336	Sulfur containing AA	1
Soybeans	204	7.2	1	14	268	Complete plant protein	2.3
Lentils	250	8.8	1.1	18	253	Sulfur containing AA	1.3
Tofu	284	10	1.3	20	189	Complete plant protein	1.3
Tempeh	306	10.8	1.4	22	265	Complete plant protein	2.4
Edamame	318	11.2	1.4	22	265	Complete plant protein	1.2
Seitan	408	14.4	1.8	29	270	Complete plant protein	no data
Buckwheat	755	26.6	3.3	53	516	Complete plant protein	0.4
Quinoa	567	20	2.5	40	555	Complete plant protein	0.5
Millet	748	26.4	3.3	53	683	Lysine, threonine	0.8
Amaranth	500	17.6	2.2	35	552	Complete plant protein	no data
Einkorn	145	5.1	0.6	10	218	no data	no data
Emmer	227	8	1	16	200	Lysine	0.3
Spelt	411	14.5	1.8	29	445	No data	no data
Kamut	411	14.5	1.8	29	454	Lysine	0.8
Almonds	227	8	1	16	575	Methionine, Cysteine	2.1
Peanut butter	68	2.4	0.3	5	470	Methionine, Cysteine	3.9
Hemp seeds	57	2	0.3	4	160	Lysine	0.7
Pumpkin seeds	132	4.6	0.6	9	433	Complete plant protein	3
Beef 15% fat	73	2.4	0.3	5	157	Complete protein	1.7
Chicken	91	3.2	0.4	6	100	Complete protein	3.3
Pork	73	2.4	0.3	5	152	Complete protein	1.9
Milk 2% fat	567	20.0	2.5	40	284	Complete protein	0.8
Eggs	188	6.4	0.8	13	291	Complete protein	2
Fish (tuna)	141	4.8	0.6	10	179	Complete protein	3.2

T: tablespoon. Combining protein-rich, plant-based foods will be the best strategy in obtaining all amino acids if partially or fully replacing animal-based foods.
